# Discovery of the World’s Smallest Terrestrial Pteridophyte

**DOI:** 10.1038/s41598-018-24135-2

**Published:** 2018-04-12

**Authors:** Mitesh Patel, Mandadi Narsimha Reddy

**Affiliations:** Bapalal Vaidya Botanical Research Centre, Department of Biosciences, Veer Narmad South Gujarat University, Surat, Gujarat India

## Abstract

*Ophioglossum* L. commonly known as “adder’s tongue fern”, has been of great interest due to the highest number of chromosomes in any organism so far known in biological world. Here, a new species of adder’s tongue fern has been discovered and reported from Western Ghats of India. It is prominently distinct from the other known taxa in Ophioglossaceae family. Phylogenetic analysis of three chloroplast DNA (cpDNA) regions (*trnL-F*, *rbcL* and *psbA-trnH*) unambiguously designate this adder’s tongue fern as the distinct lineage and is sister to the clade containing *O*. *parvifolium* and *O*. *nudicaule*. *Azolla caroliniana –* an aquatic fern (average size, 0.5*–*1.5 cm), is the smallest fern on the earth. Our discovery discloses a new species of adder’s tongue fern and ranking it among the smallest terrestrial fern in the world, attaining an average size of only 1–1.2 cm.

## Introduction

The Linnaean genus *Ophioglossum*, species of which are commonly known as “adder’s tongue fern” - owing to the shape of the spike, is the worldwide genus of eusporangiate fern family Ophioglossaceae of order Ophioglossales. Adder’s tongue ferns are too well recognized to cytologist and evolutionary ecologists for their small size with the highest chromosome number (*O*. *reticulatum* L. with 2n = 1440) in any organism so far known in biological world^1^. Historically, species concept in *Ophioglossum* is greatly misunderstood due to the simple morphology of the genus, which results in the lack of morphological characters upon which species are separated from each other^2^. Instead of lacking morphological characters, *Ophioglossum* species shows plenty of variation due to the higher number of chromosomes due to polyploidy and therefore several biotypes (morphological variants) are reported which also posed a number of problems for taxonomists^3,4^. However, species demarcation in *Ophioglossum* has been challenging, with the reported number of ~46 of species^5–10^. The prevailing problems on the genus taxonomy necessitate the genus revision with detailed morphological, palynological and by molecular methods. Genus revision is needed, because earlier taxonomic identifications were based solely on morphology and not on palynological and molecular analysis.

In India, 14 species of *Ophioglossum* has been reported, of which majority of the species are reported from the Western Ghats regions, one of the diversity hotspot in India and worldwide^11–20^. Despite a rich species diversity, Western Ghats is still under-explored region in terms of botanicals with continous sighting and findings of new species, even genera of the pteridophytes and angiosperms^21–24^.

During a botanical expedition of Ahwa forest area, Dang District, Gujarat (Western Ghats region), India we found few unusual small size of *Ophioglossum* species with very short spike, fairly close to *Ophioglossum parvifolium* Grev. & Hook. in general morphology but differing from it and other small size *Ophioglossum* species in several important characters. From morphological and spore structure comparison, we determined that the collected specimen is well-distinguished from other related small size *Ophioglossum* taxa. However, molecular phylogenetics study of three chloroplast DNA (cpDNA) regions (*trnL-F*, *rbcL* and *psbA-trnH*) unambiguously designate this adder’s tongue fern as the distinct lineage and supported the position of the taxon as a new species. Furthermore, we representing the new collected *Ophioglossum* species as the world’s smallest terrestrial pteridophyte which is very minute in size, only 1–1.2 cm in comparision to an aquatic fern *Azolla caroliniana* (average size, 0.5*–*1.5 cm)^25–27^.

## Results

### Taxonomy

#### Ophioglossum malviae

**Figure 1:**
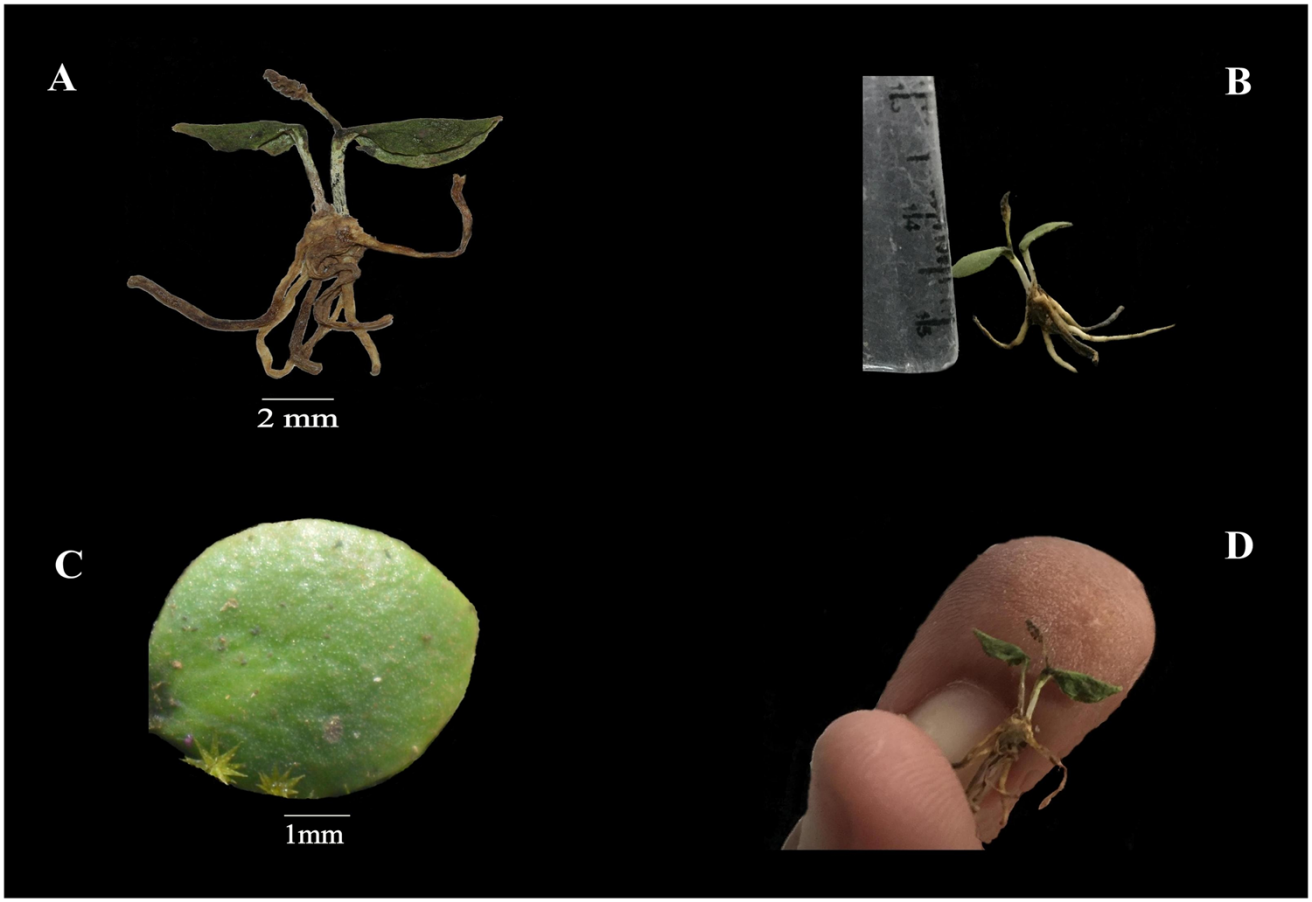
*O*. *malviae* sp. nov. (**A**,**B** and **D**) Entire plant (**C**). Tropophyll size, shape and venation.

**Figure 2:**
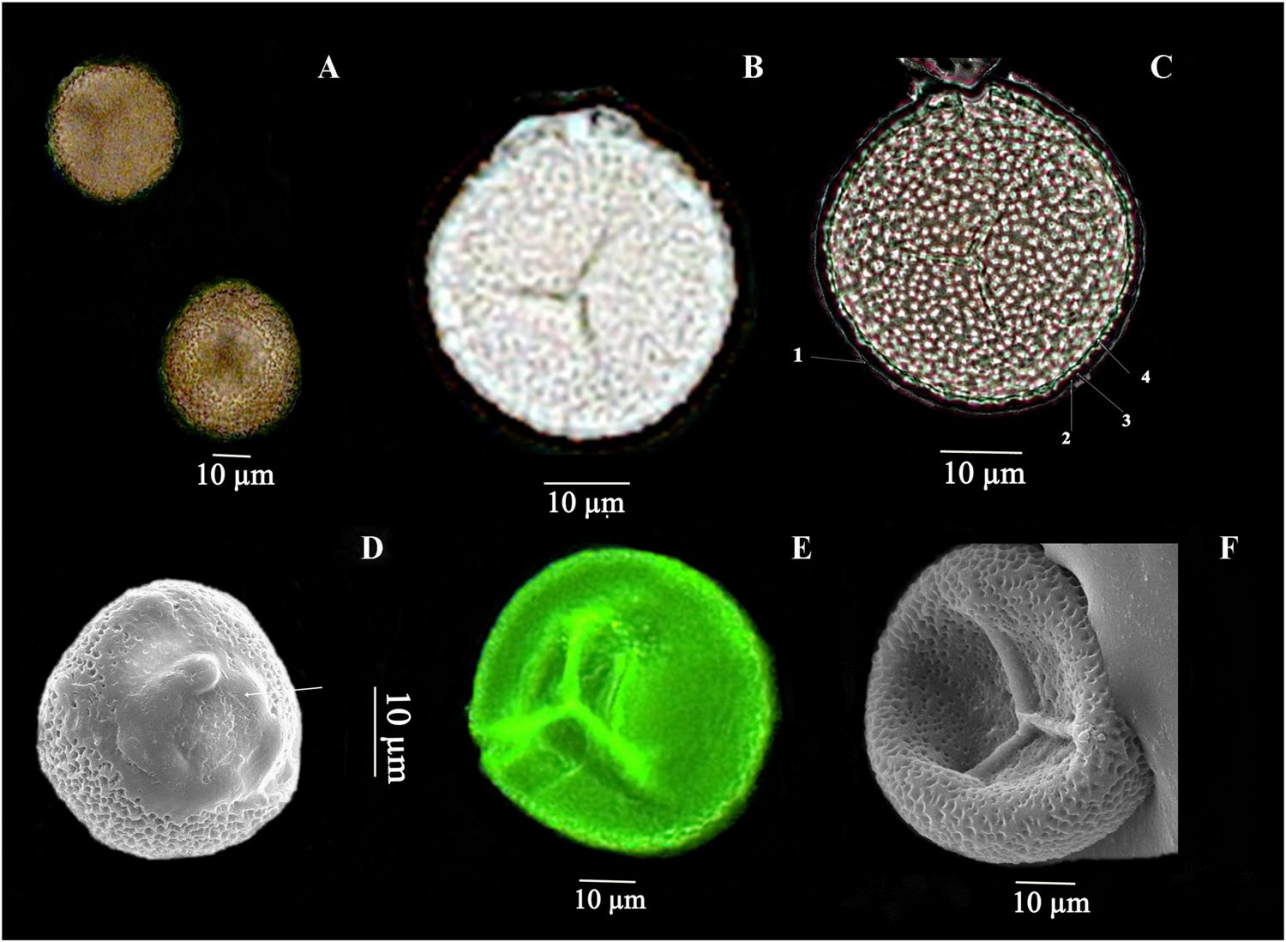
Spore structure of *O*. *malviae* sp. nov. (**A**). Spores – dorsal, ventral views in light microscopy (**B**). Spore has triradiate mark in light microscopy (**C**). Four layers of spore 1). Perine, 2). Ectexine 3). Endexine and 4). Intine in light microscopy (**D**). SEM image of whole spore from dorsal side covering with perine layer (**E**). Spore with triradiate mark in fluorescence microscopy (**F**). SEM image of spore from ventral side.

Mitesh Patel & Mandadi Narsimha Reddy sp. nov. (Figs 1 and 2).

#### Holotype

Blatter Herbarium in St. Xavier’s College, Mumbai, India (BLAT 112082!).

#### Type locality

Jakhana village (20°37′36.79′′N, 73°44′31.47′′E, altitude ~471 m), Dang district, Gujarat, Western Ghats, India.

#### Etymology

This species is named after a lady Malvi Surti who inspire the first author for pursuing research in this field.

#### Diagnosis

*O*. *malviae* sp. nov. is unique among species of this genus by its very small size and spike, spores with outer perine layer and unique type of stomata in which the marginal cells of lower epidermis form dome like papillae.

#### Description

Whole plant is minuscule, 1–1.2 cm in height; rhizome small, subglobose, tuberous, brown in colour, 0.4–0.6 cm in length, 0.2–0.3 cm in diameter; few 1–3 cm long brownish white (7–8 in numbers) roots emerges from the mid part of the rhizome; common stalk subterranean, 0.3–0.6 cm long, white in colour (Fig. 1A); rhizome bears trophophyll in set at its apex, parallel or marginally upward from the ground; trophophyll minute, 0.6–0.8 cm long, 0.4–0.6 cm wide, ovate to obovate in shape, obtuse at apex, cuneate at base, margin entire, thick and fleshy in texture, without midrib, yellowish green in shading (Fig. 1C); fertile spike inserted on adaxial side of trophophyll under trophophyll lamina, slightly yellowish green when young but brown on dry, 0.4–0.6 cm long; fertile apex very short, 0.2–0.4 cm long, 0.1 cm wide, ending with minute sterile tip; sporangia small, yellow, oppositely arranged, 4–8 in number on either side. Spores under light microscope (LM) are trilete tetrahedral type, globose, small in size (32–40 µm); presence of triradiate mark (laesurae) in the middle which is not extending up to the margins (Fig. 2A,B). In scanning electron microscope (SEM), laesural arms are crassimarginate, less wavy and jointed up to the middle of the proximal cavity (Fig. 2F). Spores are covered with four layers; outer spore wall layer forms a distinct separated layer that may appear as a loose sack is known as perispore layer (perine) (Fig. 2D); below the perispore layer, thick, reticulate ectexine and endexine layers are present; below exospore layer, inner most endospore layer is present (intine) (Fig. 2C). The distal face is granulose to verrucate in LM but in SEM the depressed aeroles are united in to negative reticulation. In the distal face, sculpture is coarsely microrugulate-microreticulate.

#### Reproductive period

August–September.

#### Distribution and ecology

India – Gujarat state, Dang district, Jakhana village. The species grows in grassy area along with mosses on the grasslands of Jakhana village at an altitude of ~471 m, located in Ahwa forest division.

#### Conservation status

Currently, about 12 plants of *O*. *malviae* sp. nov. were found in the type locality. However, this area is poorly explored for the Pteridophytes diversity. Therefore, an assumption that the other population might be distributed around this area and more further explorations are needed to determine its full range distribution, the species should be considered as data deficient for now.

### Species recognition

**Figure 3:**
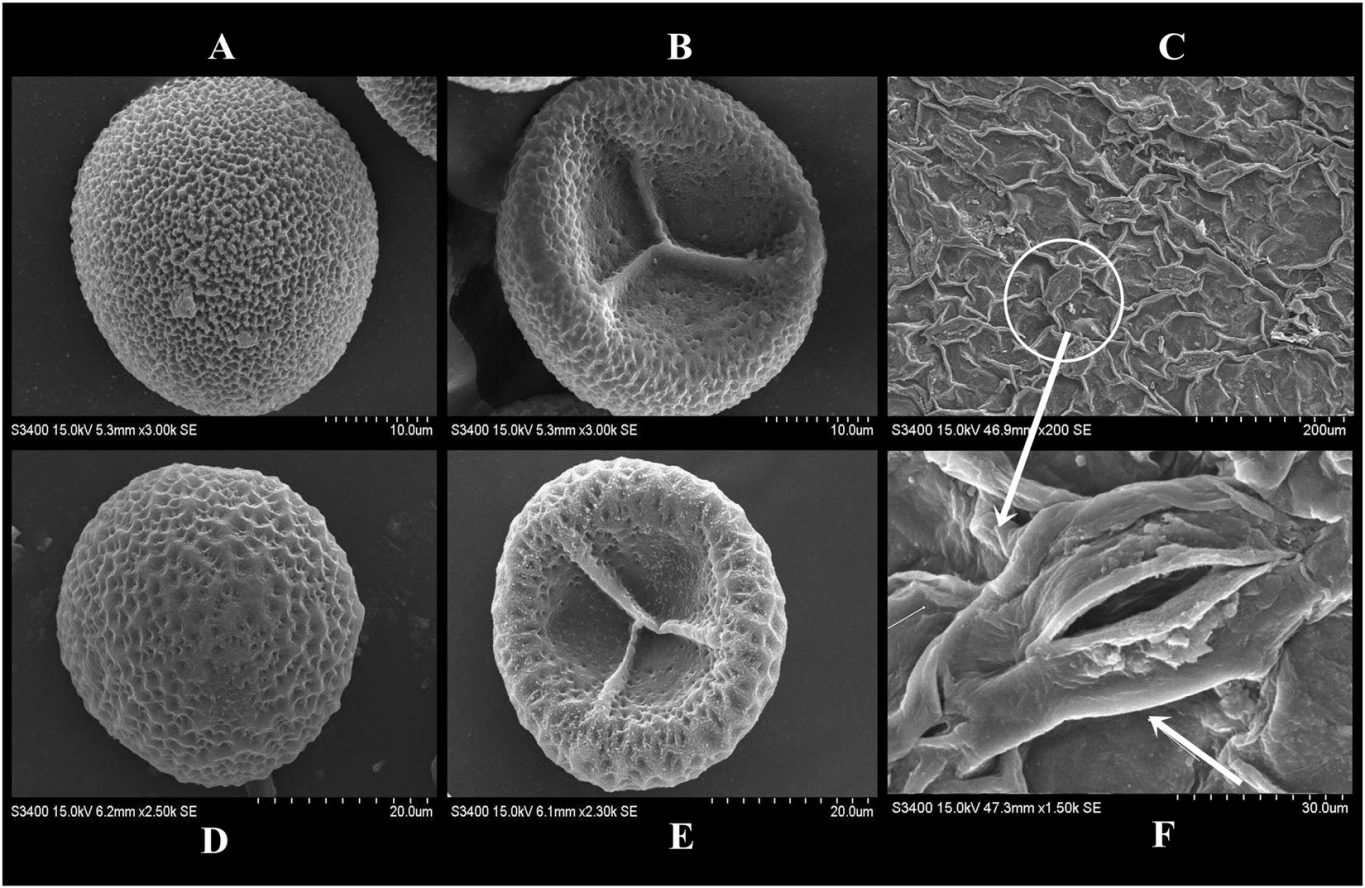
Comparision of spore structure under SEM. (**A**) *O*. *malviae* sp. nov. distal face (**B**). *O*. *malviae* sp. nov. proximal face (**C**). SEM analysis of surface of dried trophophyll *O*. *malviae* sp. nov. with distribution of distinct types of stomata (**D**). *O*. *parvifolium* distal face (**E**). *O*. *parvifolium* proximal face (**F)**. Enlargement of stomata showing dome like papillae.

*O*. *malviae* sp. nov. is unique among species of this genus by its very small size with very different spore structure and unique stomata. Vegetatively, *O*. *malviae* sp. nov. is fairly close to *O*. *parvifolium* in such features as thick trophophyll with entire margin and obscure venation which are parallel or marginally upward from the ground. However, *O*. *parvifolium* differs from *O*. *malviae* sp. nov. by its larger size (up to 10 cm), slender underground rhizome giving rise to thin, fleshy roots which often bud adventitiously to form new plants, in numbers of trophophylls (one or two) with elliptic or ovate-elliptical shape, very fine fertile stalk up to 4 cm long, strobilli up to 1 cm long consisting up to 8–16 sporangia^28^. Furthermore, trophophyll apex of *O*. *parvifolium* is acute to apiculate and base is cordate whereas, apex is obtuse and base is cuneate in *O*. *malviae* sp. nov. Common stalk is subterranean in both species although white and short in *O*. *malviae* sp. nov. whereas white at base and pale green at above in *O*. *parvifolium*. Spore structure of *O*. *parvifolium* is completely different from the spores of *O*. *malviae* sp. nov. as a major difference is the presence of an outer thick layer (perine) in spores of *O*. *malviae* (Fig. 2D) which is absent in spores of *O*. *parvifolium*. In contrast, the spores of *O*. *parvifolium* showed thickened and moderately broad wavy laesural arms in the centre of proximal cavity, that become slightly raised and extending approximately to the equator (Fig. 3E). In the distal face, *O*. *parvifolium* spores have flat and wider areas (Fig. 3D), whereas *O*. *malviae* sp. nov. spores have deeper webbed mesh with pentagonal depressions (Fig. 3A). Moreover, both species are different in epidermal mesh alignment under SEM analysis of surface of dried trophophyll. *O*. *malviae* sp. nov. showing epidermal mesh aggregations to form ridges and furrows (Fig. 3C) whereas *O*. *parvifolium* showing broken interwoven undulating epidermal mesh forming cover areas. However, in the trophophyll of *O*. *malviae* sp. nov., distinct types of stomata are present in which the marginal cells of lower epidermis form dome like papillae (Fig. 3C,F). As of now, such type of stomata were observed only in one species of family Ophioglossaceae, which is *Helminthostachys zeylanica*^1,29–32^. In this regard, the new species *O*. *malviae* sp. nov. is unique among its congeners having such dome shape stomata.

**Figure 4:**
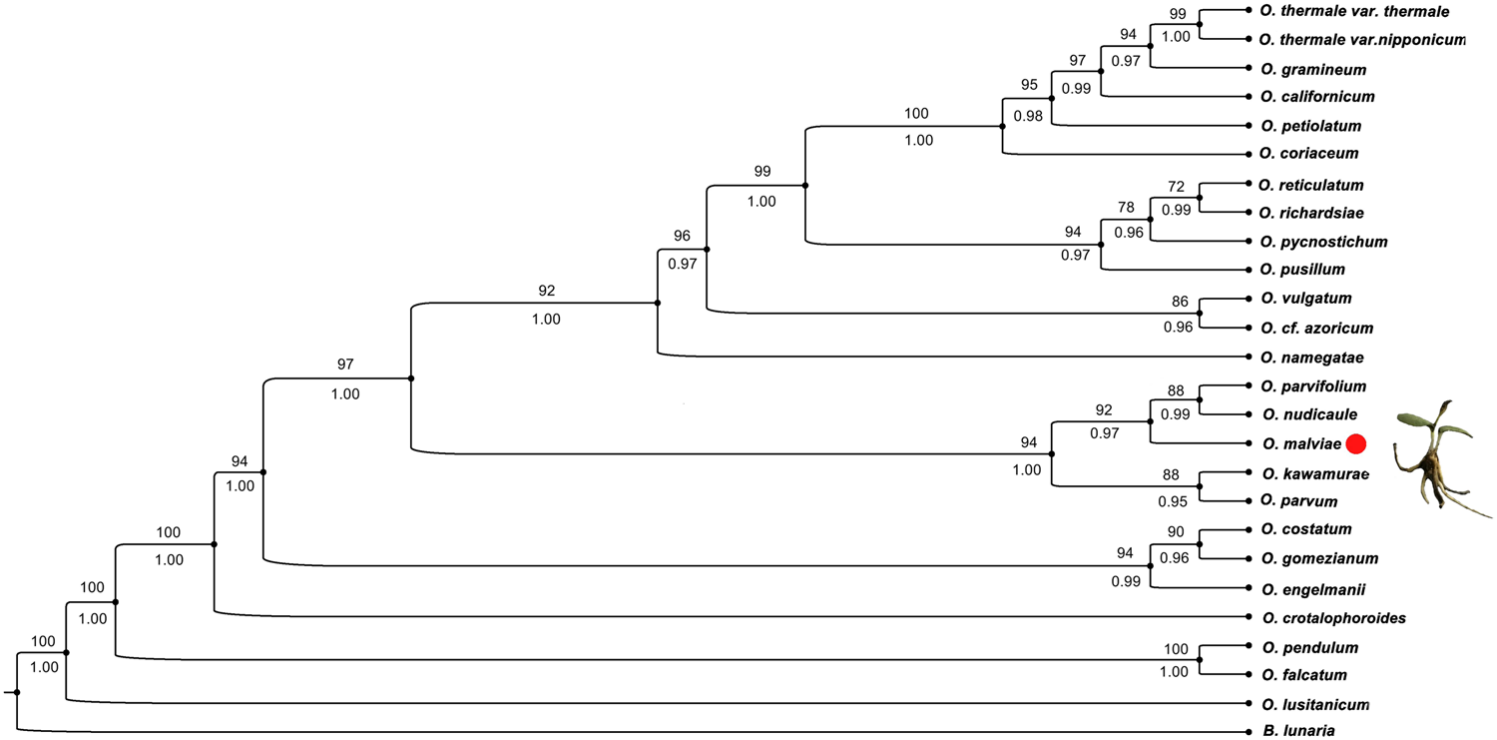
Phylogeny of *Ophioglossum* species based on concatenated *rbcL*, *trnL-F*, *and psbA-trnH* data by using maximum likelihood analysis. ML bootstrap percentages (BP) are above branches and Bayesian posterior probabilities (BPP) are below the branches.

However, our molecular results of *trnL-F*, *rbcL* and *psbA-trnH* sequence data support the status of *O*. *malviae* sp. nov. as a distinct species. It clearly and precisely indicates that it forms a sister clade (92% BS, 0.97 BPP) with a clade containing *O*. *parvifolium* and *O*. *nudicaule* (Fig. 4).

### Phylogenetic relationships and genetic divergence

Significant sequence variances between *O*. *malviae* sp. nov. and other congeneric species were seen, ranging from 1.5% to 36.0% for *psbA-trnH*, 13.3% to 64.5% for *trnL-F*, and 56.7% to 59.6% for *rbcL*. Based on the molecular phylogenetics analysis, it is established that *O*. *malviae* sp. nov. is falls within the genus *Ophioglossum*. The concatenated alignment of three chloroplast genes (*trnL-F*, *rbcL* and *psbA-trnH*) contained 49 terminal dataset with 3325 characters. 21 likelihood trees of 2468 steps (consistency index, CI = 0.56, RI = 0.68) were generated by ML heuristic analysis. Topological comparison of ML and Bayseian are almost similar but they differ in ML bootstrap value and posterior probability number. *O*. *malviae* sp. nov. represent a distinct lineage and is sister to the clade containing *O*. *parvifolium* and *O*. *nudicaule* (Fig. 4).

## Discussion

Members of *Ophioglossum* are well known not only to possess large amount of DNA, more than of most evolved angiosperms but some of its species display the extremes^33–35^. Species concept in *Ophioglossum* is greatly misunderstood historically and has posed numerous complications for taxonomists due to the lack of morphometric characters upon which species are defined. Since, high likelihood of homoplasy is tricky and difficult in the genus *Ophioglossum*, morphology based species reports are known to be problematic^36^. Moreover, simple morphology and small size of *Ophioglossum* species poses challenges in identifying them from their related species. In the present study, a new species in the genus *Ophioglossum* has been identified and differentiated from all other known species using morphological, palynological and molecular methods. High degree of phenotypic distinct lineage and genetic divergence from the clade containing *O*. *parvifolium* and *O*. *nudicaule*, we described a new species (*O*. *malviae*) to which we have designated it as the smallest terrestrial pteridophyte of the world.

In ecology and evolutionary biology research, the most imperative element in the advancement and improvement of biodiversity management strategies is species delimitation. However, in many plant groups, studies on species diversity is still poorly acknowledged, especially for those areas having exceptionally huge biodiversity and aboriginal such as Western Ghats of India.

The new species, *O*. *malviae* sp. nov. is reported from the Western Ghats of India which is home of more than 320 species of pteridophytes apart from Himalayas in the country^37^. The diversity of Pteridophytes of India appears to be well documented. However, many new species of Pteridophytes have been described from the country in recent years^38–40^, which highlight the need for more dedicated surveys across the country, specially in the less explored regions. Systematics & taxonomy of Indian Pteridophytes remains largely unattended and is in need of revision after incorporating detailed morphological, palynological as well as molecular data which will inevitably result in many more discoveries like this.

## Methods

### Morphological observations

New plant specimens were collected from the Jakhana village (20°37′36.79′′N, 73°44′31.47′′E) of Dang District, Gujarat, India during August–September, 2016. Photographs of the plant specimens were taken from the field showing their habitat. Plant specimens were collected and transferred to the laboratory for morphological as well as microscopic analysis and for herbarium preparation at Bapalal Vaidhya Botanical Research Centre (BVBRC), Gujarat, India. The study was based on examination and comparisons of specimens collected mainly from the Western Ghats regions and Central Gujarat regions of India, herbarium specimens including type collections from BLAT as well as high resolution images from SRGH (Image No: 14642, 14307, 11416, 14934, 14942, 14932, 15210, 16494, 15235, 15332), LD, US, BNRH, NYBG, K, MICH, PH, US, SGO, LINN, P, BISH, GH, F and SI, comparisons with other *Ophioglossum* species with the help of available literature and by taking guidance of the experts in the field. Spores characters were examined & photographed by using light microscope (Axioscope A1, ZEISS, Germany) and by Scanning Electron Microscopy (SEM) at Sophisticated Instrumentation Centre for Applied Research & Testing (SICART), Vallabh Vidhyanagar, Anand, Gujarat, India.

### Molecular phylogenetic analysis

#### DNA region sampling

25 *Ophioglossum* species sequences (24 sequences of *rbcL* gene, 13 sequences of *trnL-F* gene and 10 sequences of *psbA-trnH* gene) were downloaded from GeneBank in FASTA format. Downloaded sequences of all available *Ophioglossum* species were supplemented with the newly generated sequences of *O*. *parvifolium* and *O*. *malviae* sp. nov. *Botrychium lunaria* of the same family was used as outgroup taxa.

#### Isolation, amplification and sequencing of DNA

Sterilized collected plant material was used to extract the genomic DNA by modified method of Doyle and Doyle^41^. Quantification of isolated genomic DNA was done by the method of Sambrook *et al*.^42^. Purity of isolated genomic DNA was further assessed by electrophoresis in agarose gel (0.8%). Amplification was carried out by using extracted genomic DNA as template with three chloroplast DNA (cpDNA) regions (*trnL-F*, *rbcL* and *psbA-trnH*)^43–45^. All three chloroplast regions (*rbcL*, *trnL-F*, and *psbA-trnH*) were amplified via using a single protocol. 20 μL reaction mixture contained isolated genomic DNA template (2 μL) (1:10 dilution of the extracted DNA), forward primer (1 μL), reverse primer (1 μL), 1× final concentration of ReadyMix™ Taq PCR reaction mix (Sigma) (10 μL) and nuclease free water (6 μL). The reaction was carried out in Thermal cycler (Applied BiosystemsVeriti®). PCR program was adjusted as: 94 °C for 4 minutes, 30 cycles of 94 °C for 30 seconds, 50 °C for 30 seconds, and 72 °C for 1.30 minutes, and a final elongation step at 72 °C for 10 minutes and stored at −4 °C for ∞ time. Amplified chloroplast regions (*trnL-F*, *rbcL* and *psbA-trnH*) were detected via agarose gel electrophoresis (1% agarose gel) under UV light by staining with ethidium bromide. Amplified PCR products were purified using GenElute™ PCR Clean-up kit and sequenced at Eurrofins Genomics India Pvt Ltd., Bangalore.

#### Alignment and phylogenetic analysis

Sequences of all three chloroplast genes were analyzed in BioEdit 7.2.5. To find out the common regions among all retrieved *Ophioglossum* species sequences, pairwise alignment and multiple sequence alignment (MSA) was carried out by using Clustal-W embedded in MEGA 7.0. with default settings. To evaluate congruence between different DNA regions, we analyzed each dataset (*rbcL*, *psbA-trnH* and *trnL-F*) separately to see if there is a similer topology. Incongruence Length Difference (ILD) test was also performed in PAUP* ver.4.0.a159 with 1000 replication of heuristic search^46^. Lower ILD value suggesting us to analysed three dataset in combination. Separate and combined molecular phylogenetic analyses were performed using Maximum likelihood (ML) and Bayesian inference (BI) methods.

To assess best fit model of phylogenetics analyses, jModeltest ver. 2.0 was used with the implemention of Akaike Information Criterion^47^. GTR + G model was set up as the best fit for *psbA-trnH* and *trnL-F* regions whereas GTR + I + G for *rbcL* regions. ML analyses were conducted in PAUP* ver.4.0.a159 with GTR + I + G model of nucleotide substitution using heuristic analysis of 1,000 random taxon addition using TBR (tree bisection-reconnection) branch swapping^48^. To evaluate internal node support, Bootstrap analyses (BS) were calculated by 1000 stepwise addition replicates with TBR branch swapping^49^. Bayesian inference of the phylogenetic analyses was performed in MrBayes v.3.1.2^50^ and was run for independent MCMC analyses for two parallel searches from random starting trees for 5 million generations. At every thousand generations, trees were sampled. The analysis reached at a standard split frequency <0.005, and the analysis was not continued further. Twenty five percent of trees generated were discarded as burn-in and the post-burn-in samples were used as 50% majority rule consensus tree. Sequence divergence uncorrected “p-distance” was calculated by using MEGA 7.0^51^.

### Data availability

All the generated sequences have been submitted in GenBank with accession number as MF184998, MG875321, MG875322, MG875323, MG875324, MG875325, MG875326.

## Electronic supplementary material

Supplementary figures and data
