# Supplementary material for: Discovery of the World’s Smallest Terrestrial Pteridophyte
Source: Sci Rep. 2018 Apr 12;8:5911. doi: 10.1038/s41598-018-24135-2 (PMC5897345; doi:10.1038/s41598-018-24135-2)
Supplement: Supplementary file 1 — Supplementary figures and data [file 41598_2018_24135_MOESM1_ESM.pdf]

Discovery of the World's Smallest Terrestrial Pteridophyte

Mitesh Patel & Mandadi Narsimha Reddy

Supplementary Information

Supplementary Figure S1

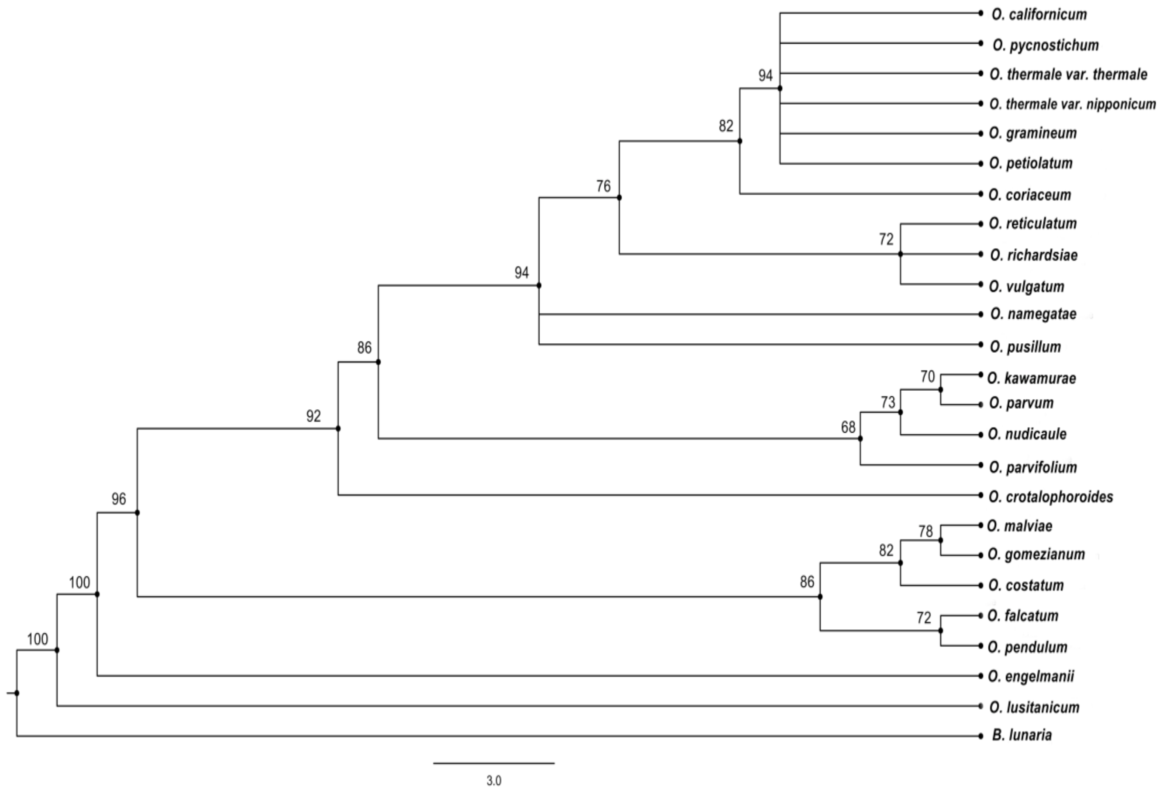

Supplementary Figure S1. Phylogeny of *Ophioglossum* species based on *rbcL* gene data by using maximum likelihood analysis. ML bootstrap percentages are above branches.

Supplementary Figure S2

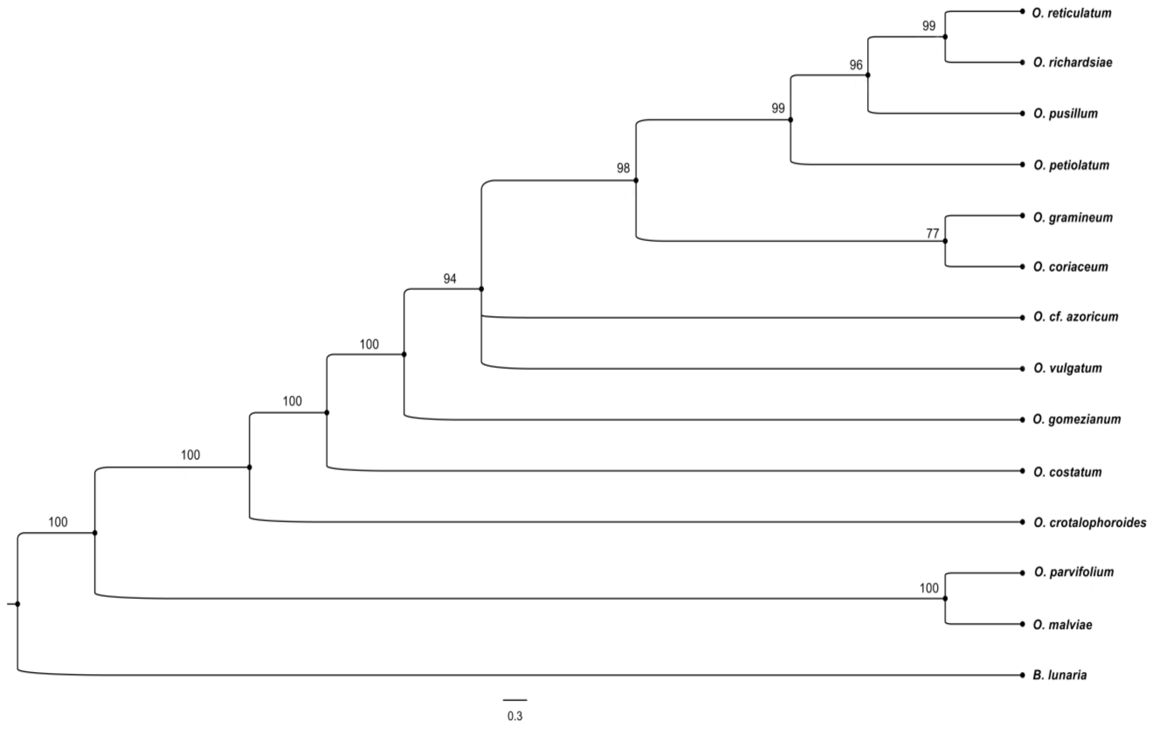

Supplementary Figure S2. Phylogeny of *Ophioglossum* species based on *trnL-F* gene data by using maximum likelihood analysis. ML bootstrap percentages are above branches.

SupplementaryFigureS3

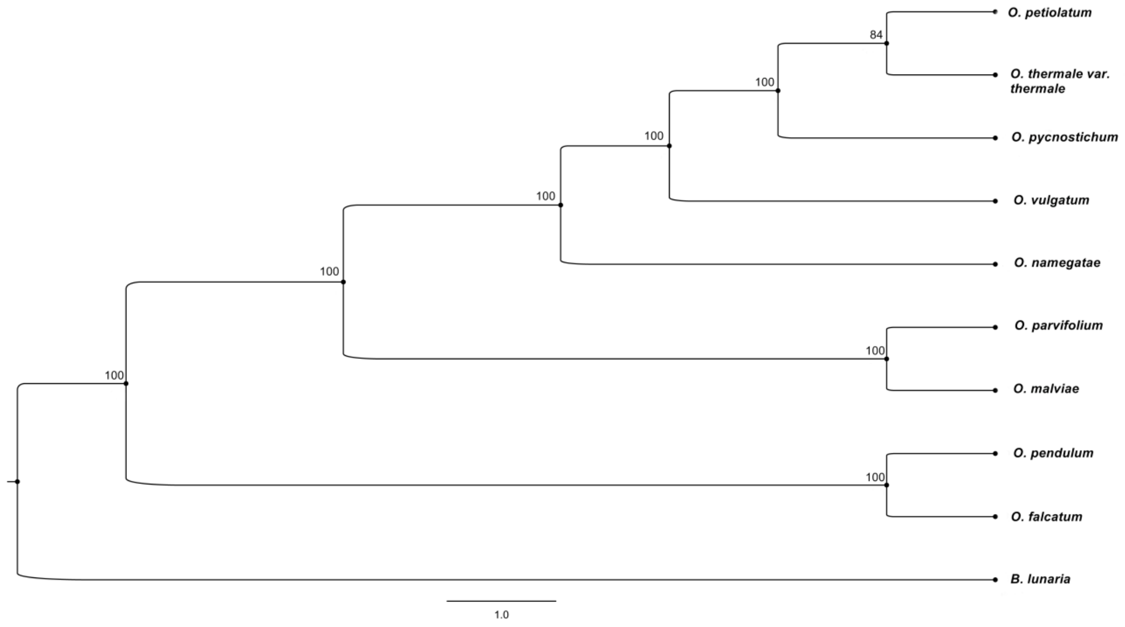

Supplementary Figure S3. Phylogeny of *Ophioglossum* species based on *psbA-trnH* data by using maximum likelihood analysis. ML bootstrap percentages are above branches.

Estimation of Evolutionary Pair wise Divergence between trnLF gene equences

Ophioglossum_malviae		0.0229	0.0339	0.0335	0.0323	0.0334	0.0334	0.0335	0.0334	0.0334	0.0334	0.0334	0.0334
Ophioglossum_parvifolium	0.1330		0.0330	0.0320	0.0318	0.0331	0.0331	0.0331	0.0329	0.0331	0.0331	0.0331	0.0329
Ophioglossum_costatum	0.5911	0.6158		0.0290	0.0273	0.0299	0.0299	0.0301	0.0304	0.0299	0.0299	0.0299	0.0304
Ophioglossum_crotalophoroides	0.6059	0.6256	0.2512		0.0262	0.0281	0.0281	0.0282	0.0287	0.0281	0.0281	0.0281	0.0287
Ophioglossum_gomezianum	0.6453	0.6502	0.2118	0.1872		0.0267	0.0267	0.0269	0.0264	0.0267	0.0267	0.0267	0.0264
Ophioglossum_gramineum	0.6207	0.6502	0.2512	0.2020	0.1872		0.0000	0.0048	0.0084	0.0000	0.0000	0.0000	0.0084
Ophioglossum_petiolatum	0.6207	0.6502	0.2512	0.2020	0.1872	0.0000		0.0048	0.0084	0.0000	0.0000	0.0000	0.0084
Ophioglossum_pusillum	0.6256	0.6552	0.2562	0.2069	0.1921	0.0049	0.0049		0.0069	0.0048	0.0048	0.0048	0.0069
Ophioglossum_reticulatum	0.6305	0.6601	0.2611	0.2167	0.1823	0.0148	0.0148	0.0099		0.0084	0.0084	0.0084	0.0000
Ophioglossum_coriaceum	0.6207	0.6502	0.2512	0.2020	0.1872	0.0000	0.0000	0.0049	0.0148		0.0000	0.0000	0.0084
Ophioglossum_cf._azoricum	0.6207	0.6502	0.2512	0.2020	0.1872	0.0000	0.0000	0.0049	0.0148	0.0000		0.0000	0.0084
Ophioglossum_vulgatum	0.6207	0.6502	0.2512	0.2020	0.1872	0.0000	0.0000	0.0049	0.0148	0.0000	0.0000		0.0084
Ophioglossum_richardsiae	0.6305	0.6601	0.2611	0.2167	0.1823	0.0148	0.0148	0.0099	0.0000	0.0148	0.0148	0.0148	

Estimation of Evolutionary Pair wise Divergence between rbcL gene equences

Ophioglossum_malviae		0.0232	0.0233	0.0233	0.0224	0.0233	0.0235	0.0230	0.0233	0.0233	0.0233	0.0232	0.0232	0.0225	0.0230	0.0229	0.0230	0.0233	0.0232	0.0233	0.0233	0.0233	0.0233	0.0233
Ophioglossum_parvifolium	0.5768		0.0110	0.0110	0.0160	0.0112	0.0160	0.0134	0.0110	0.0110	0.0110	0.0111	0.0111	0.0156	0.0128	0.0130	0.0135	0.0110	0.0111	0.0110	0.0114	0.0109	0.0112	0.0112
Ophioglossum_californicum	0.5679	0.0601		0.0000	0.0132	0.0021	0.0137	0.0092	0.0000	0.0000	0.0000	0.0040	0.0040	0.0132	0.0076	0.0078	0.0091	0.0000	0.0040	0.0000	0.0029	0.0036	0.0021	0.0021
Ophioglossum_pycnostichum	0.5679	0.0601	0.0000		0.0132	0.0021	0.0137	0.0092	0.0000	0.0000	0.0000	0.0040	0.0040	0.0132	0.0076	0.0078	0.0091	0.0000	0.0040	0.0000	0.0029	0.0036	0.0021	0.0021
Ophioglossum_falcatum	0.5947	0.1381	0.0869	0.0869		0.0133	0.0150	0.0138	0.0132	0.0132	0.0132	0.0137	0.0137	0.0067	0.0130	0.0122	0.0135	0.0132	0.0137	0.0132	0.0132	0.0132	0.0133	0.0133
Ophioglossum_coriaceum	0.5702	0.0624	0.0022	0.0022	0.0891		0.0139	0.0095	0.0021	0.0021	0.0021	0.0046	0.0046	0.0133	0.0079	0.0081	0.0093	0.0021	0.0046	0.0021	0.0037	0.0030	0.0000	0.0000
Ophioglossum_lusitanicum	0.5768	0.1425	0.0980	0.0980	0.1203	0.1002		0.0138	0.0137	0.0137	0.0137	0.0137	0.0137	0.0148	0.0139	0.0133	0.0134	0.0137	0.0137	0.0137	0.0137	0.0137	0.0139	0.0139
Ophioglossum_engelmanii	0.5657	0.1002	0.0401	0.0401	0.0958	0.0423	0.0958		0.0092	0.0092	0.0092	0.0094	0.0094	0.0136	0.0090	0.0090	0.0104	0.0092	0.0094	0.0092	0.0098	0.0097	0.0095	0.0095
Ophioglossum_thermale_var._nipponicum	0.5679	0.0601	0.0000	0.0000	0.0869	0.0022	0.0980	0.0401		0.0000	0.0000	0.0040	0.0040	0.0132	0.0076	0.0078	0.0091	0.0000	0.0040	0.0000	0.0029	0.0036	0.0021	0.0021
Ophioglossum_thermale_var._thermale	0.5679	0.0601	0.0000	0.0000	0.0869	0.0022	0.0980	0.0401	0.0000		0.0000	0.0040	0.0040	0.0132	0.0076	0.0078	0.0091	0.0000	0.0040	0.0000	0.0029	0.0036	0.0021	0.0021
Ophioglossum_namegatae	0.5679	0.0601	0.0000	0.0000	0.0869	0.0022	0.0980	0.0401	0.0000	0.0000		0.0040	0.0040	0.0132	0.0076	0.0078	0.0091	0.0000	0.0040	0.0000	0.0029	0.0036	0.0021	0.0021
Ophioglossum_kawamurae	0.5724	0.0601	0.0089	0.0089	0.0913	0.0111	0.1002	0.0423	0.0089	0.0089	0.0089		0.0000	0.0135	0.0084	0.0085	0.0098	0.0040	0.0000	0.0040	0.0051	0.0055	0.0046	0.0046
Ophioglossum_parvum	0.5724	0.0601	0.0089	0.0089	0.0913	0.0111	0.1002	0.0423	0.0089	0.0089	0.0089	0.0000		0.0135	0.0084	0.0085	0.0098	0.0040	0.0000	0.0040	0.0051	0.0055	0.0046	0.0046
Ophioglossum_pendulum	0.5969	0.1403	0.0891	0.0891	0.0223	0.0913	0.1203	0.0935	0.0891	0.0891	0.0891	0.0935	0.0935		0.0125	0.0125	0.0129	0.0132	0.0135	0.0132	0.0130	0.0131	0.0133	0.0133
Ophioglossum_costatum	0.5746	0.0891	0.0290	0.0290	0.0869	0.0312	0.1024	0.0379	0.0290	0.0290	0.0290	0.0356	0.0356	0.0802		0.0082	0.0049	0.0076	0.0084	0.0076	0.0082	0.0084	0.0079	0.0079
Ophioglossum_crotalophoroides	0.5768	0.0891	0.0290	0.0290	0.0713	0.0312	0.0935	0.0401	0.0290	0.0290	0.0290	0.0356	0.0356	0.0780	0.0356		0.0097	0.0078	0.0085	0.0078	0.0084	0.0085	0.0081	0.0081
Ophioglossum_gomezianum	0.5746	0.0980	0.0401	0.0401	0.0935	0.0423	0.1002	0.0490	0.0401	0.0401	0.0401	0.0468	0.0468	0.0869	0.0111	0.0468		0.0091	0.0098	0.0091	0.0096	0.0092	0.0093	0.0093
Ophioglossum_gramineum	0.5679	0.0601	0.0000	0.0000	0.0869	0.0022	0.0980	0.0401	0.0000	0.0000	0.0000	0.0089	0.0089	0.0891	0.0290	0.0290	0.0401		0.0040	0.0000	0.0029	0.0036	0.0021	0.0021
Ophioglossum_nudicaule	0.5724	0.0601	0.0089	0.0089	0.0913	0.0111	0.1002	0.0423	0.0089	0.0089	0.0089	0.0000	0.0000	0.0935	0.0356	0.0356	0.0468	0.0089		0.0040	0.0051	0.0055	0.0046	0.0046
Ophioglossum_petiolatum	0.5679	0.0601	0.0000	0.0000	0.0869	0.0022	0.0980	0.0401	0.0000	0.0000	0.0000	0.0089	0.0089	0.0891	0.0290	0.0290	0.0401	0.0000	0.0089		0.0029	0.0036	0.0021	0.0021
Ophioglossum_pusillum	0.5702	0.0646	0.0045	0.0045	0.0869	0.0067	0.0980	0.0445	0.0045	0.0045	0.0045	0.0134	0.0134	0.0891	0.0334	0.0334	0.0445	0.0045	0.0134	0.0045		0.0048	0.0037	0.0037
Ophioglossum_reticulatum	0.5702	0.0601	0.0067	0.0067	0.0891	0.0045	0.1002	0.0468	0.0067	0.0067	0.0067	0.0156	0.0156	0.0913	0.0356	0.0356	0.0423	0.0067	0.0156	0.0067	0.0111		0.0030	0.0030
Ophioglossum_richardsiae	0.5702	0.0624	0.0022	0.0022	0.0891	0.0000	0.1002	0.0423	0.0022	0.0022	0.0022	0.0111	0.0111	0.0913	0.0312	0.0312	0.0423	0.0022	0.0111	0.0022	0.0067	0.0045		0.0000
Ophioglossum_vulgatum	0.5702	0.0624	0.0022	0.0022	0.0891	0.0000	0.1002	0.0423	0.0022	0.0022	0.0022	0.0111	0.0111	0.0913	0.0312	0.0312	0.0423	0.0022	0.0111	0.0022	0.0067	0.0045	0.0000	

Estimation of Evolutionary Pair wise Divergence between psbH-trnL gene equences

Ophioglossum_malviae		0.0069	0.0086	0.0110	0.0279	0.0071	0.0093	0.0079	0.0278
Ophioglossum_parvifolium	0.0145		0.0086	0.0110	0.0277	0.0069	0.0091	0.0076	0.0277
Ophioglossum_petiolatum	0.0218	0.0218		0.0069	0.0274	0.0051	0.0058	0.0061	0.0279
Ophioglossum_thermale_var._thermale	0.0364	0.0364	0.0145		0.0276	0.0086	0.0090	0.0093	0.0282
Ophioglossum_pendulum	0.3600	0.3600	0.3418	0.3491		0.0274	0.0277	0.0274	0.0123
Ophioglossum_namegatae	0.0145	0.0145	0.0073	0.0218	0.3455		0.0060	0.0035	0.0279
Ophioglossum_pycnostichum	0.0255	0.0255	0.0109	0.0255	0.3491	0.0109		0.0049	0.0281
Ophioglossum_vulgatum	0.0182	0.0182	0.0109	0.0255	0.3491	0.0036	0.0073		0.0279
Ophioglossum_falcatum	0.3600	0.3636	0.3600	0.3673	0.0473	0.3564	0.3673	0.3600	
